# On the nature of the stretched exponential photoluminescence decay for silicon nanocrystals

**DOI:** 10.1186/1556-276X-6-106

**Published:** 2011-01-31

**Authors:** G Zatryb, A Podhorodecki, J Misiewicz, J Cardin, F Gourbilleau

**Affiliations:** 1 Institute of Physics, Wroclaw University of Technology, Wybrzeze Wyspianskiego 27, 50-370 Wroclaw, Poland; 2 CIMAP, UMR CNRS/CEA/ENSICAEN/UCBN, Ensicaen 6 Bd Maréchal Juin, 14050 Caen Cedex 4, France

## Abstract

The influence of hydrogen rate on optical properties of silicon nanocrystals deposited by sputtering method was studied by means of time-resolved photoluminescence spectroscopy as well as transmission and reflection measurements. It was found that photoluminescence decay is strongly non-single exponential and can be described by the stretched exponential function. It was also shown that effective decay rate probability density function may be recovered by means of Stehfest algorithm. Moreover, it was proposed that the observed broadening of obtained decay rate distributions reflects the disorder in the samples.

## Introduction

The discovery of visible photoluminescence (PL) from porous silicon and then silicon nanocrystals (Si-NCs) has stimulated a great deal of interest in this material mainly due to a number of promising potential applications, like, for instance, light emitting diodes [1] or silicon-based lasers [2]. Although the quantum efficiency of Si-NCs emission gives hope for future device applications, it remains low compared to the direct band gap III-V or II-VI materials. It is partially due to technological problems with fabrication of defect-free and structurally uniform Si-NCs samples, where nonradiative recombination sites do not play a key role in the emission process. From this point of view, the improvement of Si-NCs emission quantum efficiency remains an important challenge for further optoelectronic applications. Thus, any experimental tool that leads to information about non-uniformity of Si-NCs structures and its influence on emission properties is valuable.

The optical properties of Si-NCs can be investigated by means of time-resolved spectroscopy. This method often brings new information about disorder in the ensembles of emitters, especially in complex systems where various collective phenomena result in complicated time dependence of the experimental PL decay. Particularly, in the case of Si-NCs different authors have shown [3,4] that very often PL decay exhibits stretched exponential line shape. However, the physical origin of such behavior remains a matter of discussion. For this moment, a few explanations have been given by different authors, such as exciton migration between interconnected nanocrystals [5], variation of the atomic structure of Si-NCs of different sizes [6], carriers out tunneling from Si-NCs to distribution of nonradiative recombination traps [7] and many other [8,9]. Therefore, it is still important to gather some new experimental evidence in this field.

It should be also emphasized that in the case of stretched exponential relaxation function, the PL decay may be analyzed more thoroughly by recovering the distribution of recombination rates [10]. Surprisingly, this kind of approach is very rare with Si-NCs, especially for structures deposited by the sputtering method. Very few papers report on recombination rate distributions calculated by means of inverse Laplace transform [7] for porous silicon or by means of the maximum entropy method for Si-NCs produced by laser pyrolysis of silane [11]. Therefore, it is worth investigating the evolution of such distributions also in the case of other deposition methods.

In this work, we study the absorption properties as well as PL decays measured for Si-NCs thin films deposited by the magnetron sputtering method. It is shown that time dependence of PL may be described by the stretched exponential function. The distributions of recombination rates are calculated numerically by means of inverse Laplace transform. The influence of structural disorder on carrier relaxation kinetics is discussed.

## Experimental details

The silicon-rich-silicon oxide (SRSO) films with a nominal thickness of 500 nm used for this study were deposited onto quartz substrates by radio-frequency reactive magnetron sputtering. The incorporation of Si excess was monitored through the variation of the hydrogen rate *r*_H _= *P*_H2_/(*P*_Ar _+ *P*_H2_) from 10% to 50%. The films were deposited without any intentional heating of the substrates and with a power density of 0.75 W/cm^2^. All samples were subsequently annealed at 1,100°C for 1 h under N_2 _flux in order to favor the precipitation of Si excess and to induce Si-NCs formation.

The absorption properties were investigated by means of transmission and absorption measurements (with mixed xenon and halogen light sources). Time-resolved photoluminescence spectra were investigated by means of strobe technique with pulsed xenon lamp used as an excitation source and photomultiplier tube (PMT) used for detection. For the excitation wavelength used in our experiment (350 nm) the pulse width at half maximum was about 2 μs. To calculate the inverse Laplace transform for decay rates recovery, numerical calculations were performed using Stehfest algorithm [12] (for *N *= 14).

## Results and discussion

In our previous papers [13,14] detailed structural investigations (including atomic force microscopy, X-ray diffraction, high-resolution electron microscopy or Rutherford backscattering) of SRSO films fabricated with the same technological conditions have been reported. The main conclusion of these investigations has shown that the increase of *r*_H _used during deposition leads to increased disorder in the sample. Namely, deposition with *r*_H _= 10% favors formation of well-crystallized Si-NCs with average size of about 3 nm, whereas deposition with *r*_H _= 50% favors formation of mostly amorphous Si nanograins with size less than 2 nm.

**Figure 1:**
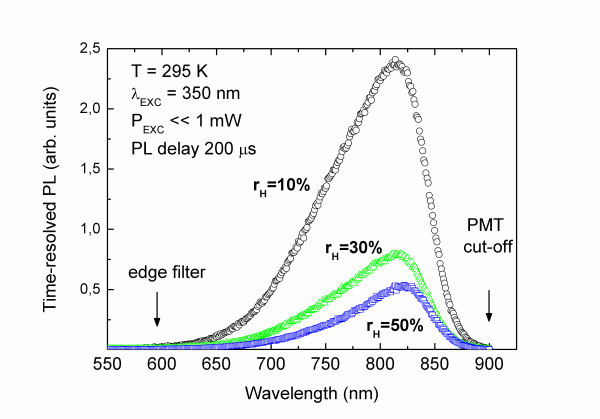
**The time-resolved PL measured for samples with various r_H _(10%, 30% and 50%)**. The nonsymmetrical emission band shape is due to the cutoff of PMT detector.

Figure 1 shows time-resolved PL spectra measured for samples with *r*_H _= 10%, 30% and 50%. In each case, the broad emission band centered at around 1.5 eV may be observed (the nonsymmetrical emission band shape is due to the cut-off of PMT detector). What is more, the PL intensity significantly drops after increasing *r*_H _from 10% to 30% and then decreases only slightly. The lack of PL shift with *r*_H _variation indicates that the observed emission rather cannot be attributed to the quantum confinement effect. It may, however, be attributed to some emission centers at the interface between Si-NCs and SiO_2 _matrix (surface states) because according to what has been shown [15], defect states localized on the nanocrystal surface may suppress the quantum confinement effect on the emission spectra. Similar effect has also been observed in one of our previous papers [16] for Si-NCs.

**Figure 2:**
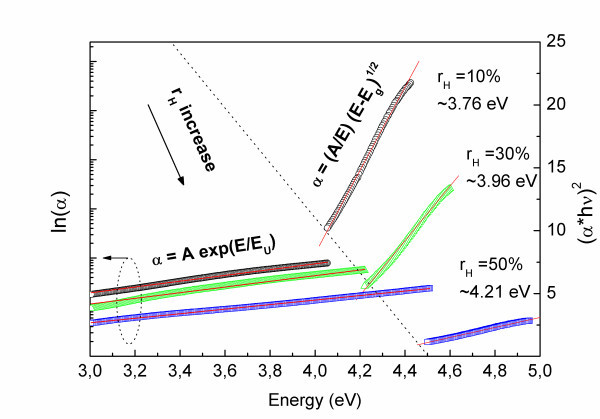
**Absorption spectra calculated on the basis of the reflectance and transmittance measurements**. The *left axis *shows long exponential tails in the absorption edge. The *right axis *shows curves used for estimating the band gap according to the Tauc formula. The blue-shift of the absorption edge may be observed with increasing *r*_H_.

Figure 2 shows the absorption (*α*) spectra calculated from reflectance (*R*) and transmittance (*T*) measurements according to the equation *α *∝ -ln(*T*/(1 - *R*)^2^) which allows us to deal with disturbing interference patterns [17]. For the higher energy part of the spectra, the Tauc formula (*αE*) = *A*(*E *- *E*_g_)^*m *^was used to estimate the optical band gap (*E*_g_). The best fit to the experimental data was obtained for m = 1/2, which corresponds to direct allowed transition. It may also be noted that the absorption edge is significantly blue-shifted from 3.76 eV for *r*_H _= 10% to 4.21 eV for *r*_H _= 50%. The observed blue-shift of absorption edge may be related to quantum confinement effect which was discussed by us in more details elsewhere [16].

What is more, below the optical band gap, the spectra shown in Figure 2 reveal long, exponentially decreasing absorption edges. Assuming that these absorption tails have amorphous nature [18] related to the structural non-uniformity of samples, they can be approximately described by Urbach equation: *α *= *C *exp(*E*/*E*_U_), where *E*_U _is the characteristic Urbach energy determining the exponential slope. Figure 2 clearly shows that *E*_U _increases with increasing *r*_H_, being of the order of hundreds of millielectron volt. Longer tails in the absorption edge for higher *r*_H _constitute another experimental evidence for stronger disorder in samples with higher *r*_H_.

To analyze the influence of structural disorder on emitters relaxation kinetic, we introduce a time-domain relaxation function that describes how system returns to equilibrium after a perturbation. Namely, after light illumination we obtain some number of emitters *n*(0) in the excited state. When we turn off the illumination the system returns to equilibrium after some time. Thus, our relaxation function may be defined as time-dependent excited emitters fraction *n*(*t*)/*n*(0).

It is well known [19] that for an ensemble of emitters, the relaxation function may be described by Laplace transform of some non-negative function *Φ*(*k*):

(1) $$\frac{n\left(t\right)}{n\left(0\right)}=\int_{0}^{\infty }\exp\left(-kt\right)\Phi \left(k\right)dk$$

In Equation 1, the function *Φ*(*k*) may be interpreted as an effective decay rate probability density function. Therefore, the decay rate *k *is in fact a positive random variable. It can be shown [20] that if relaxation of the excited emitters to the ground state may occur through many competing channels (for example the excited carriers may escape from nanocrystal to many different radiative or nonradiative centers), the relaxation function is given by stretched-exponential (Kohlrausch) function:

(2) $$\frac{n\left(t\right)}{n\left(0\right)}=\exp\left[-{\left(\frac{t}{\tau_{0}}\right)}^{\beta }\right]$$

where *τ*_0 _is an effective time constant and *β *is a constant between 0 and 1.

However, in our experiment we do not measure the relaxation function directly. Instead, we measure the number of photons *N*_Ph _emitted in a very short time period *δt *after the excitation pulse. Using a delay gate generator, we sweep the delay between moment of measurement and the excitation pulse, creating the PL decay curve. Because *N*_Ph _is directly proportional to the change of excited emitters number Δ*n *= *n*(*t*'+*δt*) - *n*(*t*'), we may define the decay of PL intensity as a negative time derivative of the relaxation function:

(3) $$I_{\text{PL}}\left(t\right)=-\frac{1}{n\left(0\right)}\cdot \frac{dn\left(t\right)}{dt}$$

Equation 3 is in fact a definition of the so called response function which determines the rate at which the relaxation function changes. Thus, in our case, the adequate form of the stretched-exponential function for PL decay is:

(4) $$I_{PL}\left(t\right)=C\cdot t^{\beta -1}\exp\left[-{\left(\frac{t}{\tau_{0}}\right)}^{\beta }\right]$$

where C is a constant.

It is also worth noting that Eq. 3 and Eq. 1 imply a more general relation between *Φ*(*k*) function and PL decay, namely:

(5) $$I_{\text{PL}}\left(t\right)=-\frac{d}{dt}\int_{0}^{\infty }\exp\left(-kt\right)\Phi \left(k\right)dk$$

which in turn leads to the following expression:

(6) $$I_{\text{PL}}\left(t\right)=\int_{0}^{\infty }k\exp\left(-kt\right)\Phi \left(k\right)dk$$

We would like to emphasize that Eq. 6 may be used to model many kinds of non-single exponential PL decays. This is an important issue, since in many papers the photoluminescence decay curve, measured in a similar way as described in the text, is modeled with the time dependence of Eq. 1. While this is a very good method to quantitatively describe the extent to which the decay is non-single exponential, it does not provide direct information about the *Φ*(*k*) probability density function.

**Figure 3:**
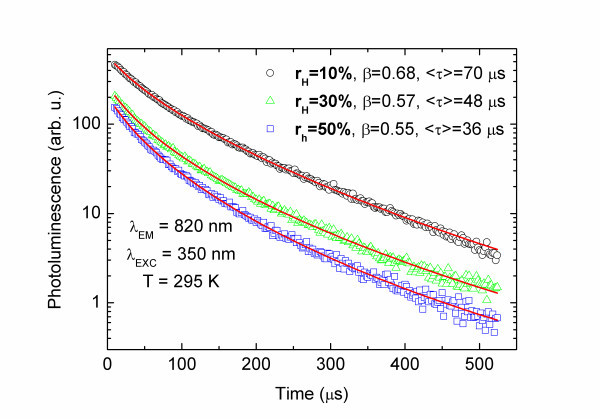
**The non-single exponential PL decays measured for samples with different r**_**H**_. The *solid line *stands for stretched exponential function fit (Eq. 1). *β *parameters and the average time constant <*τ*> are shown.

Figure 3 shows experimental PL decays measured at around 820 nm (PL peak, 1.5 eV). Hundreds-microseconds long, strongly non-single exponential decay profiles were obtained that can be well described by Eq. 4. The least-squares fit of the Eq. 4 to experimental data brings values of *τ*_0 _and *β*. Having both constants, it is possible to define average decay time constant <*τ*> in the following form:

(7) $$\langle \tau \rangle =\frac{\tau_{0}}{\beta }\Gamma \left(\frac{1}{\beta }\right)$$

where *Γ *is the Gamma function.

In the investigated case, it was found that the *β *constants were equal to 0.68, 0.57, 0.55 for *r*_H _= 10%, 30%, 50%, respectively. The average decay times <*τ*> (and *τ*_0_) were equal to 70 μs (*τ*_0 _≈ 54 μs), 48 μs (τ_0 _≈ 30 μs) and 36 μs (τ_0 _≈ 21 μs) for *r*_H _= 10%, 30%, 50%, respectively. Both parameters decrease with increasing *r*_H_. Moreover, the values of *β *and *τ *remain close to already reported results [21].

To analyze stretched exponential behavior in more details, we may recover the decay rates probability density function *Φ*(*k*). Using an analytical expression, such as Eq. 2 with *β *and *τ*_0 _taken from the experimental data fit to Eq. 4, it is possible to recover *Φ*(*k*) by means of inverse Laplace transform, solving the following equation:

(8) $$L^{-1}\left\{\frac{n\left(t\right)}{n\left(0\right)}\right\}=\Phi \left(k\right)$$

Obviously, the Eq. 7 solution depends on *n*(*t*)/*n*(0) function. In particular, for *n*(*t*)/*n*(0) given by Eq. 2, there is no general analytical solution and only asymptotic form of *Φ*(*k*) distribution may be obtained by the saddle-point method [22]:

(9) $$\Phi \left(k\right)=\frac{a\tau }{\sqrt{2\pi \beta }}{\left(k\tau \right)}^{-1-a/2}\exp\left[-{\left(k\tau \right)}^{-a}\right]$$

where *a *= *β*(1 - *β*)^-^^1 ^and *τ *= *τ*_0_[*β*(1-*β*)^1/^^*a*^]^-^^1^.

Alternatively, the inversion of Laplace transform (Eq. 8) may be computed numerically using Stehfest algorithm [12]. By comparing the numerical inversion of Eq. 8 with Eq. 9, we obtained the same results for *Φ*(*k*) distribution. In this way, we have found very good Stehfest algorithm accuracy for this class of functions. This last result may be important in a case when *n*(*t*)/*n*(0) function is a bit different than stretched-exponential, calculations with Stehfest algorithm should bring good results.

**Figure 4:**
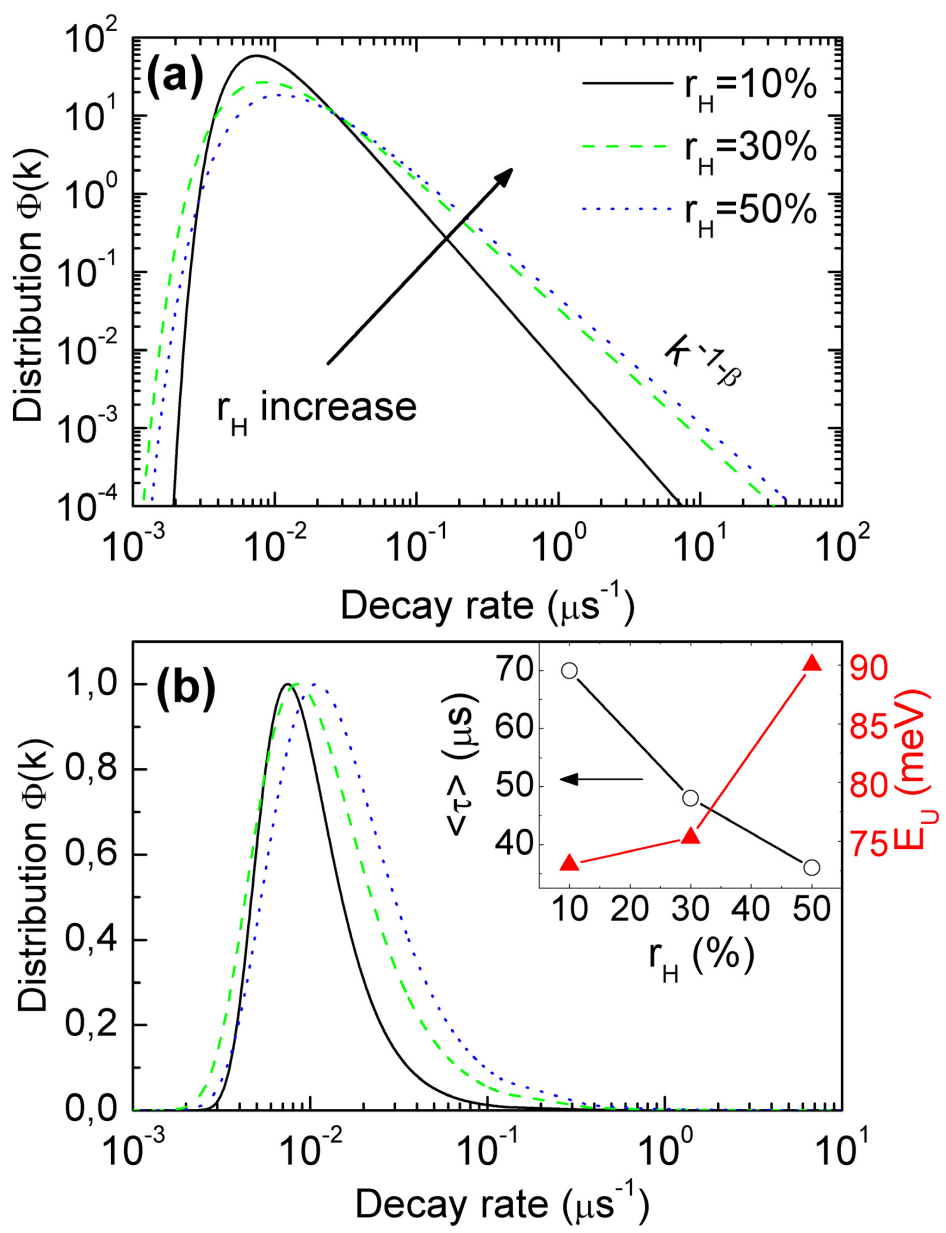
**Effective decay rate probability density functions**. Power-like dependence may be observed for higher decay rates **(a) **in log-log scale. The distribution broaden significantly with the increase of *r*_H_. Shift of the distribution is visible **(b) **together with average decay time drop and *E*_U _rise for higher *r*_H _(*inset*). The normalization in (b) was carried out in a manner exposing distributions broadening while in (a) the function *Φ*(*k*) is properly normalized probability density function.

Figures 4a,b show decay rate distribution *Φ*(*k*) calculated from Eq. 8. As expected, a power-like dependence may be observed (Figure 4a) for high values of the abscissa variable. The obtained distributions are very broad with long tails directed towards shorter lifetimes, which demonstrates the strongly non-single exponential character of decay curves. While increasing *r*_H_, the decay rate distribution *Φ*(*k*) shifts towards higher decay rates (Figure 4b). What is more, *Φ*(*k*) broaden significantly with increasing *r*_H _parameter.

To explain the observed features, it should first be mentioned that the obtained *Φ*(*k*) function provide information about both the radiative (*k*_R_) and nonradiative (*k*_NR_) relaxation rates [10]. However, a very low quantum efficiency of Si-NCs emission suggests that nonradiative processes should be predominant (*k*_NR _>>*k*_R_). This allows us to relate the changes observed in the decay rate distribution *Φ*(*k*) to the introduction of more defect states to the matrix containing Si-NCs after increasing the *r*_H _factor. These new states may act as nonradiative recombination paths for the excited carriers [7], leading to broadening of *Φ*(*k*) function and shortening of the average decay time (Figure 4b).

It is noteworthy that the above interpretation is also consistent with the rest of experimental results. As it was mentioned at the beginning, increasing *r*_H _results in higher structural disorder, which, in turn, may be the reason behind the appearance of new nonradiative states and the simultaneous increase of the Urbach energy *E*_U _(see the inset to Figure 4b). What is more, this interpretation may be also supported by our recent results [23] obtained for multilayered SRSO films with Si-NCs. In this work, we investigated samples with constant Si-NCs size co-doped with different amounts of boron. We have found that introduction of these impurities to Si-NCs environment leads to stronger deviation from single-exponential PL decays, which was interpreted as a result of appearance of new nonradiative sites. This result also correlates to the model proposed by Suemoto et al. [24], where broad distributions of decay rates were interpreted as a result of different potential barriers for carriers out-tunneling from Si-NCs to nonradiative sites.

On the other hand, it has been shown [25] that excitation may migrate from nanocrystals to light-emission centers, such as S = O bonds. Thus, if probability of such migration depends on nanocrystal structure (size or crystallinity), it is also possible that radiative recombination centers responsible for emission at 1.5 eV have a broad *k*_R _distribution (because, as structural results have shown, size and crystallinity changes with *r*_H_). Therefore, if *k*_R _was comparable with *k*_NR _then the shape of *Φ*(*k*) function could be influenced somewhat by the *k*_R _distribution. This should be especially important for samples with high quantum efficiency of Si-NCs emission (where *k*_NR _<<*k*_R _or both rates are comparable), which is not the case. Nevertheless, it is worth noting that in such case, it is also possible to obtain a broad *Φ*(*k*) probability density function and stretched-exponential PL decay.

## Conclusions

To sum up, it has been shown that PL decay of Si-NCs is strongly non-single exponential and may be described by stretched exponential function. It has been demonstrated that effective decay rate probability density function may be recovered with very good accuracy by means of numerical inversion of the Laplace transform (using Stehfest algorithm) as well as using asymptotic function. In this way, broad decay rate distributions were obtained. It has been proposed that the observed broadening of the distributions and the decrease of average decay time constant for higher *r*_H _factors is related to the appearance of more nonradiative states in the Si-NCs environment.

## Competing interests

The authors declare that they have no competing interests.

## Authors' contributions

GZ, AP and JM carried out the spectroscopic measurements as well as calculations. JC and FG designed and deposited the investigated samples. All authors read and approved the final manuscript.
